# Co-designing implementation strategies to promote remote physical activity programs in frail older community-dwellers

**DOI:** 10.3389/fpubh.2023.1062843

**Published:** 2023-03-07

**Authors:** Lorena Villa-García, Vanessa Davey, Laura M. Peréz, Luis Soto-Bagaria, Ester Risco, Pako Díaz, Kerry Kuluski, Maria Giné-Garriga, Carmina Castellano-Tejedor, Marco Inzitari

**Affiliations:** ^1^Research Group on Aging, Frailty and Care Transitions in Barcelona, Parc Sanitari Pere Virgili and Vall d'Hebron Research Institute (VHIR), Barcelona, Spain; ^2^Doctorate Program, Department of Medicine, Universitat Autònoma de Barcelona, Barcelona, Spain; ^3^QIDA, Sabadell, Spain; ^4^Population Health Sciences Institute, Faculty of Medical Sciences, Newcastle University, Newcastle upon Tyne, United Kingdom; ^5^Nursing Research Group, Parc Taulí Hospital Universitari, Institut d'Investigació i Innovació Parc Taulí (I3PT-CERCA), Universitat Autònoma de Barcelona, Sabadell, Spain; ^6^Nursing Department, Faculty of Medicine, Universitat Autònoma de Barcelona, Barcelona, Spain; ^7^Centre d'Atenció Primària Bordeta-Magòria, Barcelona, Spain; ^8^Bridgepoint Collaboratory for Research and Innovation, Bridgepoint Health, Toronto, ON, Canada; ^9^Institute of Health Policy Management and Evaluation, Dalla Lana School of Public Health, University of Toronto, Toronto, ON, Canada; ^10^Department of Sport Sciences, Faculty of Psychology, Education and Sport Sciences, Blanquerna, Universitat Ramon Llull, Barcelona, Spain; ^11^Department of Physical Therapy, Faculty of Health Sciences, Blanquerna, Universitat Ramon Llull, Barcelona, Spain; ^12^Faculty of Health Sciences, Universitat Oberta de Catalunya (UOC), Barcelona, Spain

**Keywords:** older adults, frailty, aging, mhealth, World Café, integrated care, participatory methods, co-design

## Abstract

**Background:**

The “AGIL Barcelona (AGILBcn)” community-based integrated care program is a multicomponent healthy aging intervention for frail older adults. In this context, the present study aimed to identify implementation strategies to optimize the accessibility, acceptability, and adaptability of mobile health (mhealth) interventions to enhance physical activity in frail older adults, and to prioritize action points according to their importance and feasibility, through a co-design process.

**Material and methods:**

A mixed methods approach was used. In the qualitative phase, a method adapted from the World Café was applied in 6 virtual groups to identify strategies to facilitate the virtual physical activity program. In the quantitative phase, prioritization and feasibility of the strategies was analyzed through surveys. Strategies were ranked based on priority vs. feasibility, revealing if strategies should either be: implemented first; if possible; taken into account for future consideration; or directly disregarded. The convenience sample included older adults (*n* = 7), community professionals (*n* = 9) and health professionals (*n* = 13). Qualitative data were analyzed by summative content analysis and quantitative data by nonparametric descriptive analyses.

**Results:**

A total of 27 strategies were identified and grouped into four categories: general strategies for reducing barriers; specific strategies for facilitating the use of a digital application; specific strategies for facilitating participation in virtual exercise groups; and specific strategies for facilitating external support. According to the ranking of strategies, the first ones to be implemented included: digital literacy, digital capability assessment, family technology support, weekly telephone follow-up by professionals, personalizing exercises, and virtual exercises in small groups.

**Conclusion:**

The active participation of all stakeholders enabled us to identify potential strategies for implementing person-oriented technology in physical activity programs and for engaging older adults.

## 1. Introduction

The aging of the population is accompanied by an acceleration in the incidence of disability (1). Frailty, defined as a pre-disability state of initial impairment of intrinsic capacity, is a target for interventions aimed at improving function and delaying disability (2). Multicomponent lifestyle interventions aimed at promoting healthy aging have proven to be effective in the short term (3, 4).

During the COVID-19 pandemic, social distancing protocols and the subsequent demand for community spaces led to an increase in sedentary behavior in older adults (5), contributing to the progression of frailty and disability (6). An alternative approach to traditional face-to-face physical activity interventions that has gained special momentum is the use of mobile health (mhealth) (7). Technology-based interventions appear to positively influence physical activity levels in older adults (8) and offer the potential to reach individuals on a large scale while allowing for personalized programs. Despite the availability and potential of technology for enhancing physical activity (9), barriers to its adoption and use by older adults and in different care settings remain (10, 11).

It is widely recognized that there is a significant gap between the development of evidence-based interventions for public health and health promotion and their successful and sustainable implementation (12). Approaches for promoting physical activity in older adults using mhealth present unique challenges. Currently, most physical activity promotion interventions remain limited to the experimental or pilot phase, as their continuous implementation or scale-up poses large difficulties. These include a limited understanding of implementation strategies and a failure to match these to the needs of end users.

Engaging end users in the development of health promotion interventions and the design of digital solutions incorporating elements derived from participatory methodologies, conceived within the framework of patient and public involvement (PPI), is key to achieving strategies that are contextually adapted and conducive to their sustained adoption and implementation (13). Participatory design, now known as co-design, is hypothesized to have a strong and lasting impact on health outcomes and may represent a promising strategy for addressing complex health behaviors. Co-design in this context specifically refers to patients and caregivers working collaboratively with health and allied health professionals to improve service delivery by sharing knowledge and experience (14). Its goal is to optimize the implementation of evidence-based interventions according to the priorities and preferences of all stakeholders, enabling designed solutions to achieve maximum feasibility and sustainability.

The present study is part of the +AGIL Barcelona (AGILBcn) program (15), a complex community intervention co-designed by and for frail older adults, together with primary care teams and community stakeholders. The program encompasses different aspects of health including physical activity, nutrition, emotional wellbeing, sleep hygiene, cognitive screening and stimulation, loneliness, and medication review. The AGILBcn multicomponent exercise program consists of 10 face-to-face group sessions led by a physiotherapist in a primary care setting. The program is complemented by exercises performed at home and prescribed through the publicly available ViviFrail^®^ App (16). Results showed a positive impact on physical function at 3 months (17).

Due to the COVID-19 pandemic and the ensuing challenges facing health services, pressure to redesign the program in a virtual or semi-virtual format increased. However, despite the great potential of digital technology to enhance the promotion of healthy lifestyles in older adults, a lack of specific implementation strategies could even exacerbate health inequalities, increase costs, and jeopardize implementation in routine practice.

This work aims to identify implementation strategies for optimizing the accessibility, acceptability, and adaptability of mHealth interventions aimed at increasing physical activity, within the framework of AGILBcn or similar programs, and to assess their level of priority and feasibility through a co-design process aimed at ensuring equal and equitable participation of multiple stakeholders.

## 2. Materials and methods

### 2.1. Study design

A mixed-methods study was designed, incorporating both qualitative and quantitative data and adopting a triangulation multilevel model, to elicit views from key stakeholders: older adults (OA) as end users; health professionals (HP); and professionals from the community and voluntary sector (CVS). We selected these key participants in order to assess the accessibility, acceptability and adaptability of the AGILBcn virtual program, aimed at older adults with frailty but absent or mild disability. Specific themes were addressed, including: barriers related to the “digital divide” that must be overcome, to ensure the viability of incorporating mHealth (app and virtual exercise sessions) into the program; logistics of exercising and conducting virtual exercise sessions from the homes of older adults; and monitoring, support and other factors that could affect uptake, motivation and adherence to the program.

The co-design process was carried out in two phases described below: (1) six virtual “World Café” (18) sessions (renamed as “AGIL Café” sessions) to identify implementation strategies for facilitating the deployment of the AGILBcn virtual program; and (2) evaluation of the level of priority and feasibility of the strategies identified during the AGIL Café sessions, using digital surveys.

### 2.2. Settings and participants

Participation was sought to represent the main stakeholders in the community-based multi-component AGILBcn program (15). Participants included older adults, health professionals and professionals from the community. Purposive sampling was used to identify and select key participants capable of offering a wealth of information regarding the phenomenon of interest (19, 20). Inclusion criteria for participants were:

- Older adults with no or minimal disability in performing basic daily living activities, and with no acute diseases, aged 70–90 years, and presenting at least one sign of frailty (i.e., slow gait speed, weakness, memory complaints, involuntary weight loss, or poor social support), able to participate in videoconferences, fluent in Catalan or Spanish and without speech disorders.
- Health professionals (physicians, nurses, physiotherapists, neuropsychologists, occupational therapists, or social workers) with more than 6 months of work experience in primary care or geriatric services and in complex chronic conditions.
- Professionals from the community and voluntary sector: workers from third sector services targeted at older adults (municipal or non-profit programs).

Three researchers (LMP, LS, MI) were responsible for recruitment. Potential participants were contacted either by telephone (OA, previous or potential participants in AGILBcn) or e-mail (HP and CVS), to request participation and to explain the objectives, structure and format of the sessions. HP were recruited from an intermediate care hospital and a primary care center in Barcelona and were selected for diversity in profession, work area and professional experience. CVS were recruited based on the type of community organization they worked for (e.g., civic centers, pharmacies), and professional experience related to community programs targeting older adults (e.g., programs to increase physical activity, improve digital skills, reduce loneliness).

We aimed for between 6 and 12 participants per stakeholder group and invited 13 participants to each group to ensure participation. Sample size was determined based on the capacity of the selected sample to provide information and on a criterion of information redundancy in the identification of new codes or themes (20).

Of the 13 candidates from each group who were contacted for recruitment, 6 OA decided not to participate due to health-related problems or overlapping duties (which the research team had tried to accommodate), 4 CVS declined the invitation due to work commitments, and all HP agreed to participate. Finally, 7 OA, 9 CVS and 13 HP agreed to participate. No participants withdrew from the study.

### 2.3. Data collection

#### 2.3.1. Phase 1: Procedure of AGIL Café

This study used the World Café participatory research approach (18) to facilitate structured and unstructured collaborative dialogue and knowledge generation for the resolution of common problems from the perspective of multiple stakeholders (21, 22). This method allows for obtaining the lived experience of the participants and their needs and preferences for services, dividing large groups into smaller ones while remaining part of a single, connected conversation (22). It has been used in a wide variety of settings, including the development and evaluation of health services (22) and for the improvement of care for older adults (23).

Six AGIL Café sessions (2 groups for each profile) were conducted. The decision to avoid mixed groups was made to give equal status to end users and thereby avoid the risk of reduced participation due to differentials in status and experience (24).

The AGIL Café sessions took place between December 2020 and March 2021, with a duration of 1.5 h per session, conducted in a virtual format (25). The Zoom^®^ communication platform was chosen for its video and audio quality, functionalities and simplicity. Meetings were password protected. At the time of the meeting, attendees were sent to a waiting room where identity was confirmed. Sessions were video and audio recorded. The process was guided by a multidisciplinary research team with experience in primary, geriatric care, physical activity promotion programs and qualitative research experience. In each workshop, a member of the team acted as facilitator; an additional member admitted participants to the call and helped to solve technical problems during the session (LS); two recorded ideas (VD and MI); and two others acted as observers and evaluators of the process (LMP and LV). To stimulate the conversation, the team developed a script, adapted for each group, containing main questions and subsidiary prompts (Supplementary Table 1). These were guided by study objectives, existing literature, and independent and representative feedback on understandability and comprehensiveness. Sessions were conducted in rounds (introduction followed by a round for each question). To facilitate the participation of all attendees and to prevent any single participant from monopolizing the conversation, each participant was invited to respond by the moderator, who carefully monitored responses. After each round, time was allocated to unstructured discussion. The real-time LucidChart^®^ app was used by the researchers to record and visualize ideas presented by participants using virtual “sticky notes” and graphics functions. This enabled the correction and clarification of suggestions made and permitted the continuous overview of ideas generated, facilitating reflection.

#### 2.3.2. Phase 2: Prioritization and viability of changes identified in the co-design groups: Surveys

Based on the qualitative analysis of the AGIL Café sessions (phase 1), an *ad hoc* questionnaire was developed in Catalan, consisting of 27 potentially actionable strategies for facilitating the AGILBcn virtual program, which were subdivided into 4 categories or blocks.

The questionnaire required that each item be ranked according to its perceived priority and feasibility using a 5-point Likert scale from P1/F1, representing the highest priority/highest feasibility, to P5/F5, representing the lowest priority/lowest feasibility (the range of options is described in Supplementary Table 2). A participant from each stakeholder group was asked to review the questionnaire prior to its dissemination, to identify any problems and rate its comprehensibility. The survey was conducted using the online platform LimeSurvey^®^ between May and June 2021 with a 100% response rate. The survey was distributed to HP and CVS *via* email, with information on its purpose and objectives. The survey entry screen specified how data would be used and requested informed consent. Participants could withdraw at any time before submitting their final responses.

For OA, the survey was disseminated and completed *via* computer-assisted telephone interviewing to avoid any potential difficulties from the use of online platforms. Questions were read aloud directly from the online survey, and responses were recorded in real-time in the online system. A single trained interviewer (LS) conducted all surveys from the call center of the referral hospital.

### 2.4. Data analysis

#### 2.4.1. Qualitative data

Content analysis (26, 27) was performed to identify all potentially actionable strategies raised by participants in the AGIL Café sessions using AtlasTi™, based on transcripts, field notes and visual record captured in Lucidchart™ for additional clarification. Some interpretation was required, to distinguish relevant material: two researchers worked together (LV, VD), thoroughly reviewing the material generated from each session, and carrying out analysis independently. Once finished, the codes, categories and themes were unified and agreed upon.

Once coded, frequencies and quotations were derived for all potentially actionable items and analyzed. Initially, 48 codes were identified, discussed and reviewed by the research team. Codes representing the same underlying concept were collapsed into one category, and codes were grouped into sections covering specific themes, resulting in the categorization of four umbrella categories and 27 codes. Questions for the survey were then developed to elicit views on the priority and feasibility of the proposed strategies, for practical purpose and to validating and triangulating the groups' data (28). We also analyzed the quantitative data to show the participation of stakeholders in the categories, and as such, their initial “ownership” of ideas; this provided a background to the interpretation of survey results and assisted in our appraisal of the co-design methodology.

#### 2.4.2. Quantitative data

The Likert scale results for each of the 27 items of the phase 2 questionnaire were analyzed using non-parametric descriptive statistics. We assigned numerical values to the categorical ratings for priority (P) and feasibility (F) (separately) and converted all responses into numerical scores. Values were as follows: P1/F1 −100 (highest), P2/F2 −75 (high), P3/F3 −50 (medium), P4/F4 −25 (low), P5/F5 −0 (lowest). Using these values, we calculated:

• The “priority vs. feasibility score” (PvF score), which corresponds to the average of the priority and feasibility scores, providing an estimate of the global relevance of each item.

• The difference between the mean priority and feasibility score, which gives an idea of the agreement between P and F. We included this parameter because, although a strategy might rank high overall in PvF, it might show a gap between its P and F (e.g., high P and average or low F) reflecting a lack of agreement between priority and feasibility scores.

All scores were calculated for each of the participant profiles. Data were analyzed and processed using STATA^®^ and Excel^®^.

### 2.5. Ethical and research approvals

Ethical approval was obtained from the Clinical Research Ethics Committee (CREC) of the Foundation University Institute for Primary Health Care Research Jordi Gol i Gurina (IDIAPJGol) (20/048-P) and by the Ethics Committee on Animal and Human Experimentation (Authorization Number CEEAH 5066) of the Universitat Autònoma de Barcelona (UAB). All participants received verbal and written information about the study and provided written consent for recording the sessions, using anonymized verbatim quotations in the reporting of data, and using audio, photograph and/or video recordings of the sessions in dissemination.

## 3. Results

### 3.1. Characteristics of study participants

The ages of participating OA (*n* = 7) ranged from 70 to 90 years (Table 1), in line with participants in the AGILBcn program. CVS was the most diverse in background, encompassing professionals working as part of neighborhood health plans (*n* = 2), a community project aimed at tackling loneliness (*n* = 1), a city community project for improving the situation of people in need of care and their caregivers (*n* = 1), a neighborhood civic center (*n* = 1), a community pharmacy (*n* = 1) and a foundation that assists older adults living alone (*n* = 2).

**Table 1:** Characteristics of the sample.

**Participant**	**Sex**	**Age**
OA1	Woman	82
OA2	Man	81
OA3	Woman	84
OA4	Woman	86
OA5	Woman	88
OA6	Woman	79
OA7	Woman	83
**Participant**	**Sex**	**Professionals from the community and voluntary sector**
CV1	Woman	Community Pharmacy
CV2	Man	Technician developing neighborhood health plans
CV3	Woman	Foundation that helps elderly people living alone
CV4	Man	Foundation that helps elderly people living alone
CV5	Man	Community project to tackle solitude
CV6	Woman	Technician developing neighborhood health plans
CV7	Woman	Departament Promoció persones grans
CV8	Woman	A city community project to improve the situation of people in need of care and their caregivers
CV9	Woman	Neighborhood Civic Center
**Participant**	**Sex**	**Profession**
HP1	Woman	Computer systems expert
HP2	Man	Physiotherapist
HP3	Woman	Doctor
HP4	Woman	Doctor
HP5	Woman	Neuropsychologist
HP6	Woman	Doctor
HP7	Woman	Nurse
HP8	Woman	Nurse
HP9	Woman	Nurse
HP10	Man	Doctor
HP11	Woman	Doctor
HP12	Woman	Occupational Therapist
HP13	Woman	Social Worker

From the participating HP (*n* = 13), the most represented professions were medical doctors (5) and nurses (3). Other allied HP included a psychologist (*n* = 1), a physiotherapist (*n* = 1), an occupational therapist (*n* = 1), and a social worker (*n* = 1). We also included in this group an expert in healthcare information and communication technology (*n* = 1).

### 3.2. AGIL Café results

The results have been structured into 2 themes: (1) Suggested strategies that were, on the surface level: (i) actionable (to some degree) on the short term; (ii) within the boundaries of the project and, (iii) within the scope of influence of the actors involved, either at an individual or institutional level; and (2) Priorities for change that were wider in scope than the project and could not be actioned upon on the short term.

This paper focuses on the first theme. Our data coding and categorization process revealed four main categories and 27 codes of potentially actionable strategies (Table 2). The results are organized into four categories: (1) general strategies for reducing barriers to older adults participating in a virtual program (2) specific strategies for facilitating the use of a digital application to prescribe and teach individualized exercises and to monitor progress; (3) specific strategies for facilitating the participation of older adults in virtual exercise groups, performed at home *via* group video calls; and (4) specific strategies for facilitating external support, if needed.

**Table 2:** Description and frequency of the main strategies (codes) suggested by the participants for implementing a virtual exercise program, grouped into four main categories.

**Codes**	**All (*n* = 29)**	**CvS (*n* = 9)**	**HP (*n* = 13)**	**OA (*n* = 7)**
**General strategies for reducing barriers to older adults in a virtual program**				
Assess digital capacity	8	0	8	0
Conduct educational meetings in advance to train and educate in the use of technology	3	0	3	0
Provide simple, paper-based educational materials on how to use technology	6	0	3	3
Inform family on the selected solutions to reinforce the use of technology	0	0	3	0
Assess the need for external support with technology and facilitate it, if necessary	5	1	4	0
Provide continuous technological support	6	4	2	0
**Specific strategies to facilitating the use of a digital application to prescribe and teach individualized exercises and to monitor progress**				
Provide feedback from a healthcare professional by phone, on individual progress	5	0	2	3
Use gamification techniques	3	0	2	1
Implement a formal digital “expert user” program (support by “peer champions”)	3	2	1	0
Create a simple educational video on how to use technology and share it *via* chat	1	0	0	1
**Specific strategies for facilitating the participation of older adults in virtual exercise groups, performed at home** ***via*** **group video calls**				
Establish preferred platform for video calls	1	0	1	0
Implement systems for sending reminders with dates, *via* chat apps or phone calls	1	0	1	0
Inform caregiver or support volunteers about the class schedule	1	0	1	0
Provide a variety of physical exercises	3	0	0	3
Limit the size of the virtual group	3	0	0	3
Incorporate music in the sessions	9	0	0	9
Involve older adults in the co-design of the sessions	2	1	1	0
Provide feedback from a healthcare professional by phone, on group progress	5	0	2	3
Create peer-to-peer/group messaging in the chat application	3	1	2	0
**Specific strategies for facilitating external support if needed**				
Recruit local volunteers offering digital support	6	6	0	0
Organize peer support for technology	4	3	1	0
Develop an intergenerational technology literacy program with students	0	2	0	0
Custom referrals to local support services	9	5	4	0
Use local groups or volunteer networks to provide technological support	11	6	5	0
Prioritize any agency/group known to the individual as external support	9	8	0	1
Develop a formal support plan agreement/ social prescription of the program	13	7	6	0
Train primary care staff in support options and referral processes	2	1	1	0

CvS, professionals from the community and voluntary sector; HP, Health professionals; OA, older adults.

The numbers in the table represent the number of participants that suggested that particular strategy.

Below, we provide an overview of how each stakeholder profile contributed to the set of actionable implementation strategies generated, with examples; observations are presented in accordance with the Consolidated group exercise for Reporting Qualitative Research guidelines (COREQ) (29).

#### 3.2.1. Category 1: General strategies for reducing barriers to older adults in a virtual program

Strikingly, 85% (*n* = 17) of the strategies generated in this category came from HP, with lengthy discussions on concerns of lack of digital literacy among OA, much of which was from direct experience (Table 2). Early assessment of digital capacity was considered something that should become standard practice.

“In geriatrics we are very used to using scales for everything, if there is a scale for a pre-measurement of their digital skills, it should be part of the holistic assessment of the person” (HP 3: Woman, health professional, Doctor).

Moving beyond this, pre-intervention face-to-face contact with end users and caregivers was perceived as key to guaranteeing an understanding of the program and of potential barriers for each person (and his/her caregiver), and to devising person-centered strategies to trying to reduce them.

“There is a need for an initial visit, where they are accompanied. This is how to introduce physical activity, technology and stimulate involvement and motivation” (HP 4: Woman, health professional, Doctor).

All participating groups described the need for digital training programs, although suggestions varied. HP underlined the benefits of paper manuals, in combination with further scheduled contact during the intervention period:

“At the time of seeing them, if you can, reinforce and review their ability to interact and use ‘the app'... To do this, I have created written support, a mini-manual, with steps adapted to the person's ability” (HP 5: Woman, health professional, neuropsychologist).

#### 3.2.2. Category 2: Specific strategies for facilitating the use of a digital application to prescribe and teach individualized exercises and to monitor progress

Relatively few suggestions (*n* = 12) were made on how to improve the accessibility and viability of using a digital application for personalized exercise plans for all groups (Table 2). End users' reactions to indirect support mechanisms such as training videos and paper guides were mixed. Some participants found using a video guide rather than written instructions more appealing, and vice versa. One person described following exercises at home alone with a paper or video guide as “sad”. Support *via* trained expert users in digital literacy was mentioned by professionals, in line with expert patient programs to promote autonomy and self-care in people with chronic pathologies, but end users were unsure about this when it was suggested by the researchers. Most of the end users said, however, that they would be concerned about whether or not they were “getting it right”. This was tied to a belief that performing the exercises incorrectly would result in not obtaining the desired improvement. They felt more confident if a professional followed up on the activity at regular intervals to “control results; if you have done it, or if you have not” (OA 2: Man, older adult). Weekly follow-up by a health professional was suggested only by a minority from this group; others spoke about capacity issues. Game elements, such as rewards and leveling up, were mentioned by a minority of HP and older adults, but signs of improvement were viewed by end users as the primary motivation:

“As long as you see that [doing exercise like this] helps...that you notice that you're getting better...” (OA 2: Man, older adult).

#### 3.2.3. Category 3: Specific strategies for facilitating the participation of older adults in virtual exercise groups, performed at home *via* group video calls

Older adults were most vocal in suggesting ways of making virtual exercise groups more accessible and appealing to them (68%) (Table 2). The option of participating in virtual exercise groups was seen by end users as preferable to being prescribed physical exercise alone through videos or a worksheet. Many said that, ultimately, face-to-face groups were more desirable to them for social interaction. Still, some mentioned that virtual groups might be easier because of mobility concerns, fear of falling, pain restricting mobility and fear of (COVID-19) infection:

“For me, the greatest difficulty would be not being able to do it in the neighborhood without having to take public transport” (OA 4: Woman, older adult).

Limited group size was raised by many as necessary to ensure personalized attention. Some had had negative experiences attending large and overcrowded group exercise classes, targeted generally at their age range. Music featured heavily in the discussion. They felt that they would find it much more enjoyable and easier to perform if the accompanying music was adapted to the exercises to be performed.

#### 3.2.4. Category 4: Specific strategies for facilitating external support if needed

In contrast to the first category, which was formed largely from HP input, “external support” mechanisms were predominantly raised by CVS, reflecting their work (Table 2). As with the first category, almost none of the strategies from this domain user were shared by our older adults' representatives. This was unsurprising, as all had some level of family support available for digital literacy:

“..... I see my daughter every morning, I will tell her to teach me” (OP 1: Woman, older adult).

While there was much agreement on drawing on community support networks to assist people without family support, the potentially actionable strategies offered were diverse. Local groups or established support networks featured more frequently than the more loosely defined “local volunteers”, with emphasis placed on making the most of existing resources (whatever they may be) at the neighborhood level. To this extent, CVS representatives encouraged mapping local resources including spaces, such as libraries and civic centers, which offered meeting points and internet connection. Many of the participants from the third sector spoke of the longer-term purpose of empowering older adults and fostering social relations. The needs of the virtual AGILBcn program, for ensuring accessibility and promoting adherence should be subsumed under other endeavors:

“I think it would also be important to have the option of having two older people together who can receive the training, so we encourage something that is also very important... peer socialization” (CVS 5: Man, professional from the community).

### 3.3. Prioritization process

The AGIL Café sessions generated a large number of potentially actionable strategies (Table 2). There was also an obvious clustering of suggested by the professional group (HP vs. CVS). This created challenges for appraising the value and adaptability of possible strategies to optimize the accessibility, acceptability and adaptability of the virtual program. Consequently, the survey, eliciting views on prioritization, offered the participants the opportunity to evaluate all proposed strategies.

### 3.4. Priority vs. feasibility score

According to overall PvF score (Table 3), the top ten most valued strategies were related to: (a) improving group exercise through videoconference (limited group sizes, personalized exercises, choice of a preferred platform, reminders for the classes, and music); (b) general ways to overcome technological barriers (meetings to prepare and train users of technology, identification of a support person, shared information with family about the technology employed before the start of the program, and assessment of the need for external support with technology and facilitate it, if necessary and (c) the use of Apps (periodic follow-up calls to check on the use of the App and the progression of the program).

**Table 3:** Comparison of priority (P) vs. feasibility (F) score (PvF) for each suggested strategy, and differences between average P and F for each item of the questionnaire.

	**Blocks (4)**	**PvF Score**				**Difference (P, F)**			
		**All**	**CVS**	**HP**	**OA**	**All**	**CVS**	**HP**	**OA**
n		29	9	13	7	29	9	13	7
Limit the size of the virtual group to facilitate personalized attention	Virtual groups	81	64	87	93	−3	0	−6	0
Provide a variety of physical exercises to be tailored to the individual	Virtual groups	75	54	83	89	−1	8	−7	0
Conduct educational meetings in advance to train and educate in the use of technology	General	74	65	80	73	−4	8	−17	4
Establish a preferred platform for video calls	Virtual groups	73	69	77	70	−6	−11	−6	0
Implement systems for sending reminders with dates *via* chat apps or phone calls	Virtual groups	73	58	85	70	1	6	−3	3
Provide continuous technological support	General	71	55	74	84	−12	0	−29	4
Incorporate music in the sessions	Virtual groups	71	51	77	84	1	−8	8	4
Inform family on the selected solutions to reinforce the use of technology	General	69	56	76	71	−8	6	−17	−7
Assess the need for external support with technology and facilitate it, if necessary	General	69	53	69	89	−14	0	−31	0
Provide feedback from a healthcare professional by phone, on the individual progress	App	69	65	65	82	−11	−8	−19	0
Assess digital capacity	General	68	65	77	55	−4	−3	−19	4
Provide simple, paper–based educational materials on how to use the technology	General	67	57	74	68	8	14	10	0
Provide feedback from a healthcare professional by phone, on the group progress	Virtual groups	67	51	72	78	−7	−3	−14	0
Create a simple educational video on how to use technology and share it *via* chat	App	66	64	68	64	6	17	2	0
Use gamification techniques	App	66	55	76	61	0	12	−10	0
Involve older adults in the co–design of the sessions	Virtual groups	64	36	83	66	−10	−12	−15	4
Inform caregiver or support volunteers about the class schedule	Virtual groups	63	51	68	68	−2	−3	−2	0
Use local groups or volunteer networks to provide technological support	External support	63	50	73	61	−7	8	−19	0
Recruit local volunteers offering digital support	External support	62	59	63	61	−9	−3	−19	0
Create peer-to-peer/group messaging in the chat application	Virtual groups	61	62	65	50	6	3	11	0
Custom referrals to local support services	External support	61	54	70	54	−11	0	−23	0
Prioritize any agency/group known to the individual as external support	External support	60	55	73	43	−9	−4	−16	0
Organize peer support for technology	External support	57	53	59	59	−16	−17	−21	−4
Implement a formal digital “expert user” program (support by “peer champions”)	App	55	66	47	60	−15	0	−22	−19
Develop an intergenerational technology literacy program with students	External support	55	38	65	61	−8	−9	−12	0
Train primary care staff in support options and referral processes	External support	54	50	68	36	−14	−7	−22	0
Develop a formal support plan agreement/ social prescription of the program	External support	53	55	62	33	−5	18	−21	3
All		65	56	72	66	−6	< 1	−12	< 1

Items are ordered from highest to lowest PvS. Blocks (4) refer to the 4 categories into which the 27 strategies are grouped in Table 2.

PvF score, priority and feasibility score; CVS, professionals from the community and voluntary sector; HP, Health professionals; OA, older adults. PvF score is calculated as the averaging the mean priority and feasibility scores; the difference is between the mean priority and feasibility score. For the PvF score, the higher score, the best “compromise” between P and F, whereas for the Difference, a value close to 0 indicates the highest coherence between the 2 construct.

Average PvF score for all stakeholder groups tended to smoothen the contribution of each group, compounded by the uneven number of participants in each; thus, we also present the results stratified by groups (Table 3). Maintaining a person-centered approach was a priority, so it is important to note that 9/10 items prioritized by the users were concordant with the top ten from the overall ranking. Finally, CVS scores were systematically lower on all items, although the rank of priority was similar that of the other groups.

### 3.5. Differences between priority and feasibility scores

When looking at the difference between priority and feasibility (Table 3), the top three actions in terms of feasibility (group sessions through videoconference with a low number of participants and a high personalization of exercises, as well as setting-up a meeting specifically for preparing for the use of technology), seemed coherent in terms of both priority and feasibility. In contrast, the assessment of the need for external support, the identification of a support person and the provision of weekly follow-ups with users of digital apps correspond to actions with apparent lower feasibility than priority. End users tended to express the highest coherence in the feasibility of the actions with higher priority. On the other hand, HP had the lowest confidence in the feasibility of actions with the highest priority.

### 3.6. Prioritization of solutions by means of a prioritization matrix

**Figure 1:**
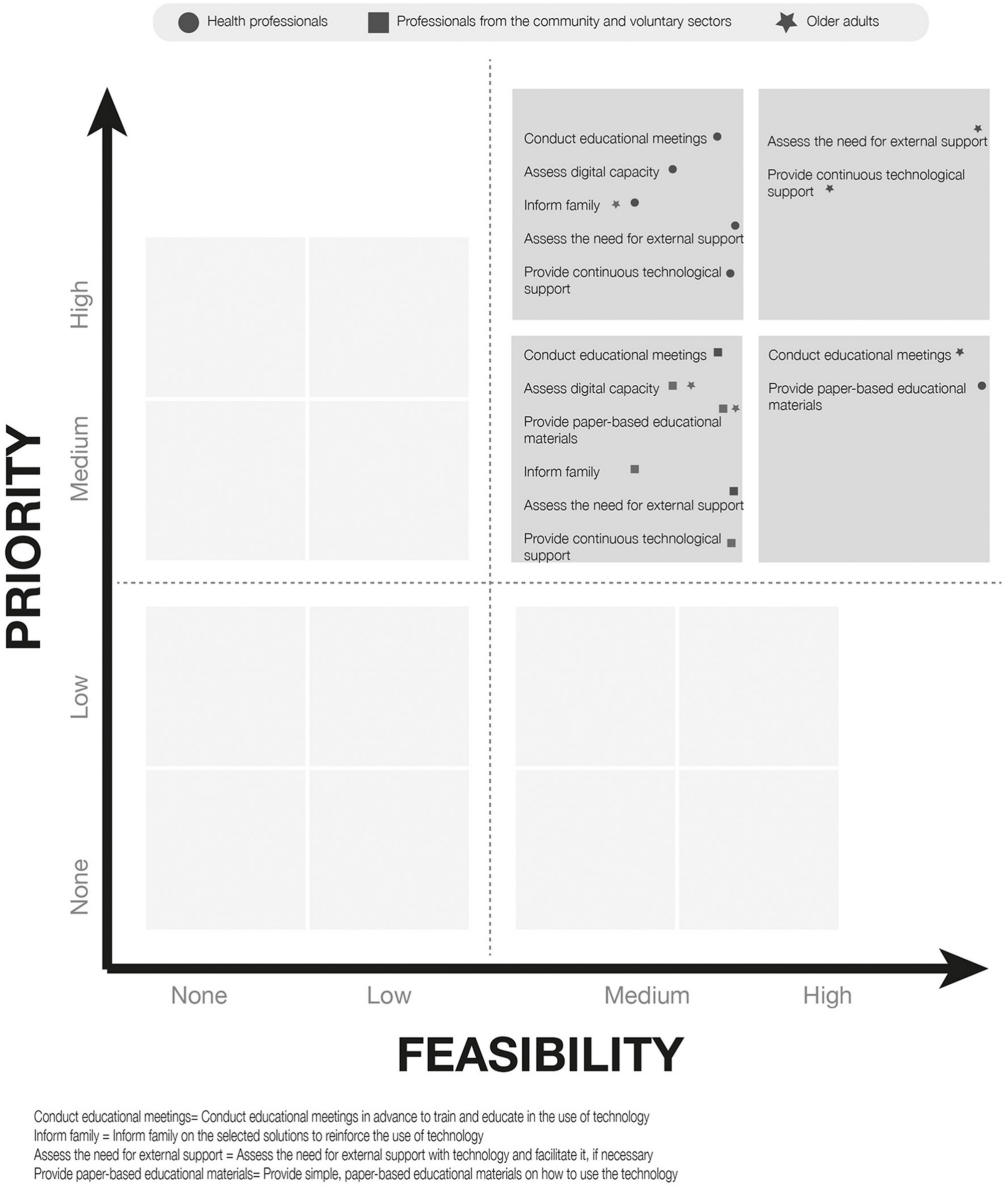
Mapping of responses to general strategies to reduce digital barriers to older adults.

**Figure 2:**
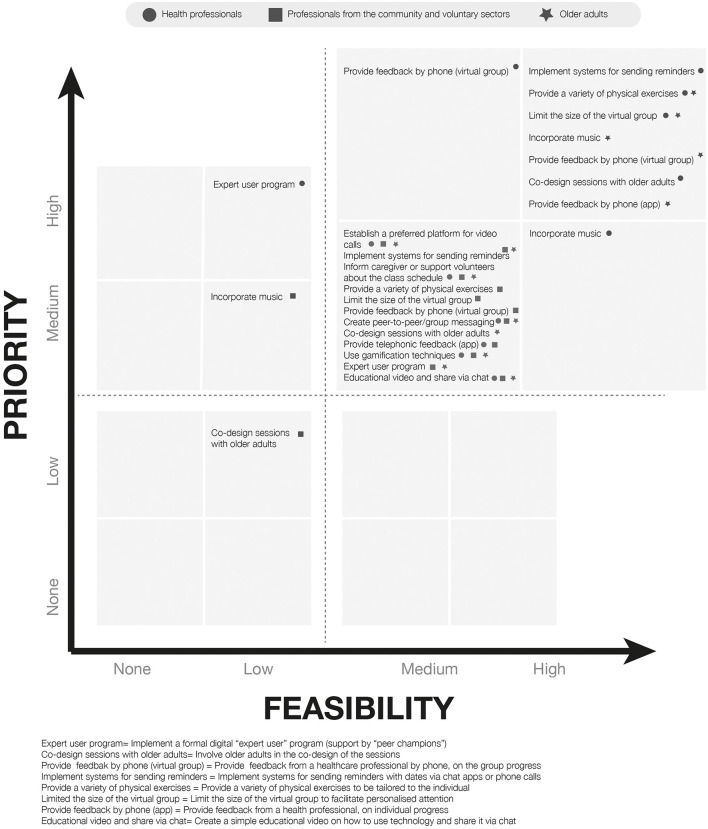
Mapping of responses to specific strategies to facilitate the use of physical activity App and specific strategies to facilitate the participation of the older adults in virtual exercise groups performed at home during group video calls.

**Figure 3:**
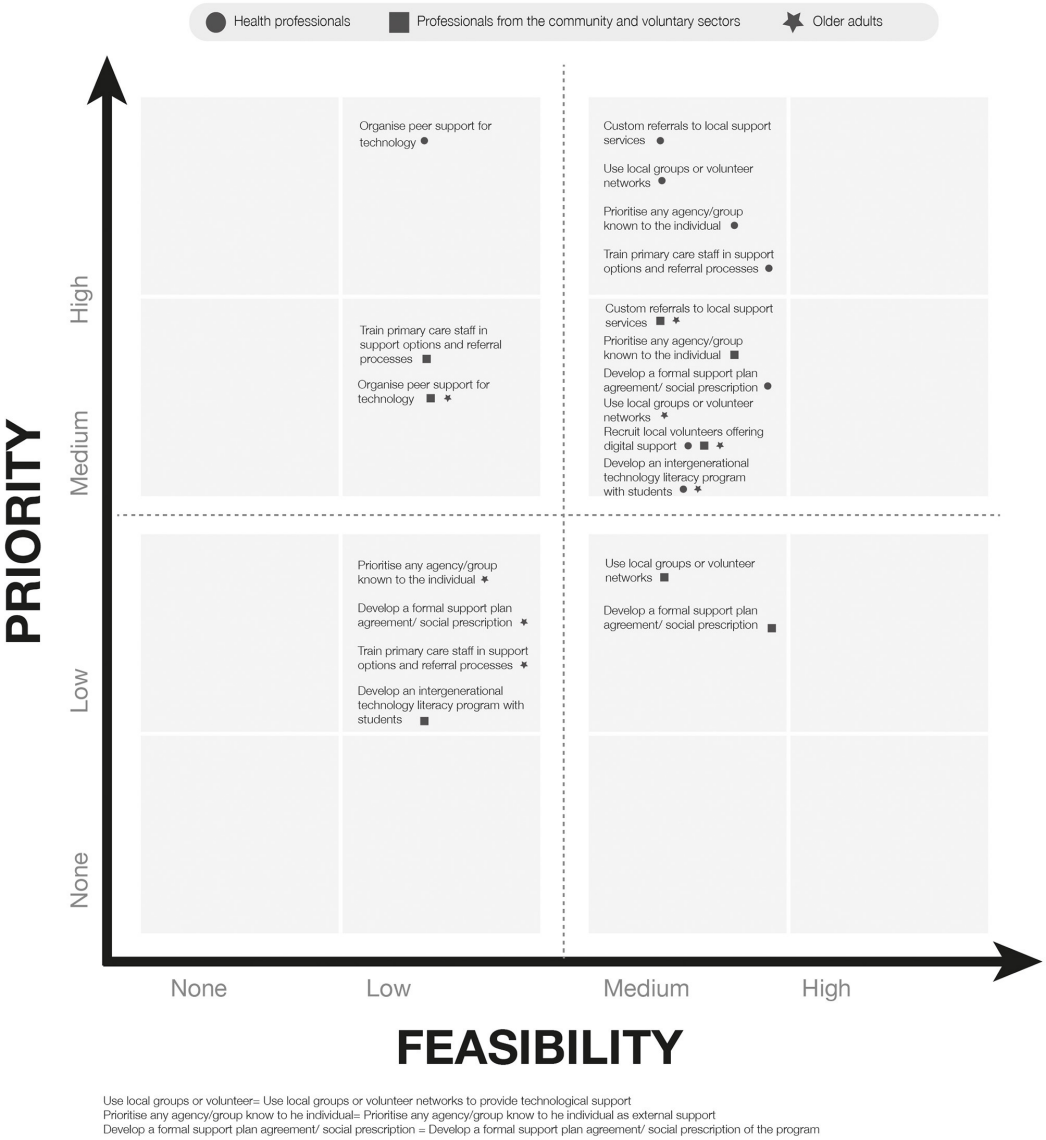
Mapping of responses to specific strategies to facilitate external support.

The answers to the questionnaire were then plotted in a 4 × 4 matrix categorizing the combined priority and feasibility response for each item ranging from top priority-top feasibility to no priority-no feasibility, according to the priority and feasibility scores for each item (score 75–100 = top, 50–74 = medium, 25–49 = low and 0–14 = no priority or feasibility (Supplementary Table 2). The responses for each sub-section of the questionnaire were plotted to create visual maps (Figures 1–3).

**Figure 4:**
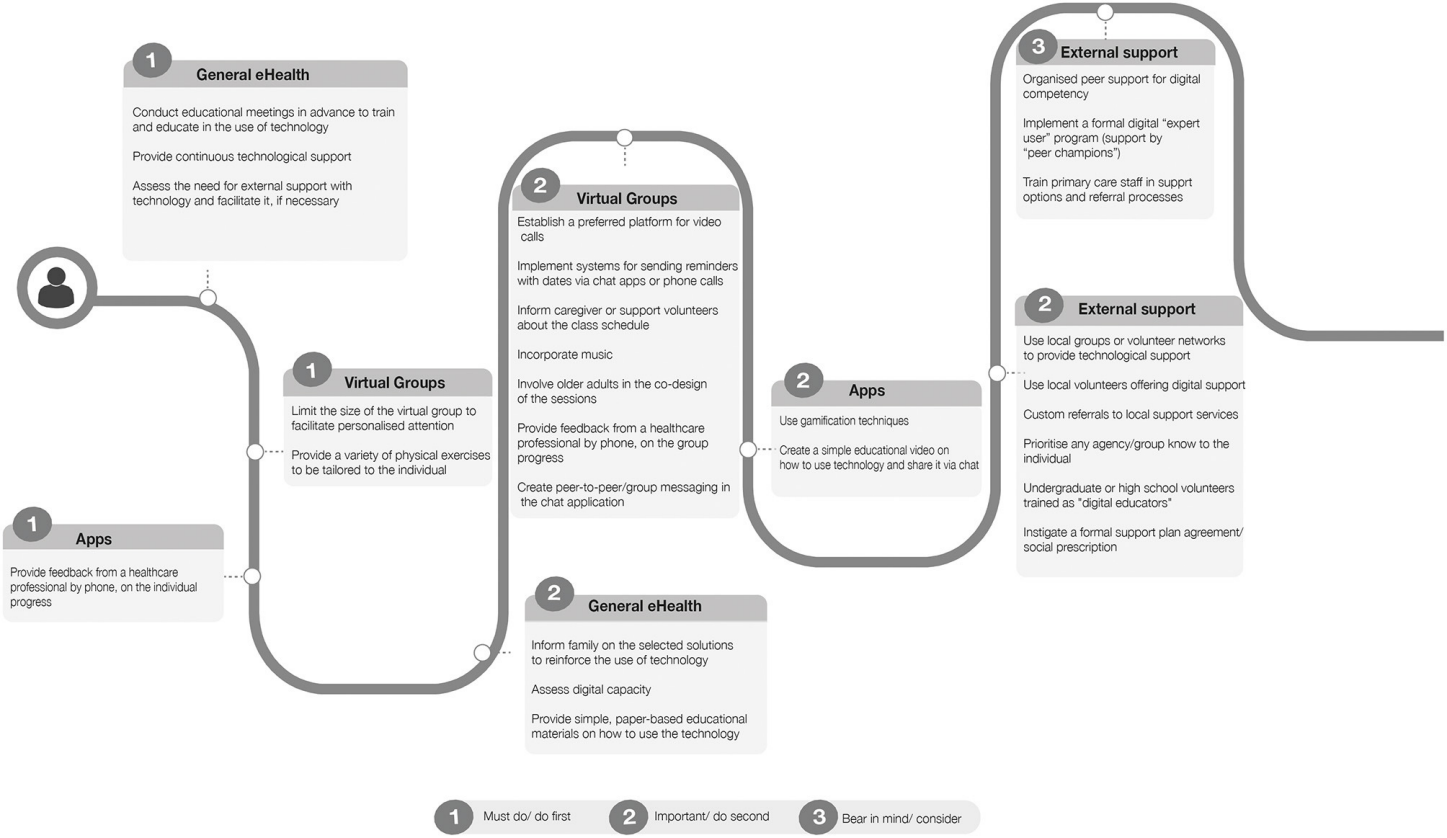
Graphic representation of the main suggestions from all groups, plotted along an ideal timeline, from the preparation to the execution of the mHealth solution to foster physical activity.

This procedure allowed us to map the proposed solutions in terms of their importance and feasibility or practical need for action: must do, do first; important, do second; do if possible; bear in mind/ consider; and do not consider. Figure 4 represents the different proposed solutions as a “visual journey” of tasks that should be considered on a timeline, from the beginning to the end of a virtual exercise program, with each task coded according to this 4-category priority matrix. This map might add value in terms of a meaningful and workable way of looking at the results in order to guide the adaptation process, in accordance with the data generated from the co-design process. In particular, it ensures that the data on both priority and feasibility have an even influence on the results and that the results of each group have an even influence, despite the differences among groups in the respective number of participants. Consequently, one of the six items that appear as “must-do” does not match with the top six items according to the PvF score (weekly follow-up telephone calls to those using digital apps, substituting virtual exercise class reminders). The other “must do” items are coherent with the PvF score ranking (set up a preparation meeting, identify a support person for technology, assess the need for external support, limit the group size and personalize the exercises during videoconference groups). No solution was classified as “do if possible” or “do not consider”.

## 4. Discussion

Through the +AGIL Café sessions, 27 possible strategies were suggested to adapt a multicomponent program aimed at enhancing physical activity in older adults, based on accessibility, acceptability and adaptability. These were grouped into 4 categories: general strategies for reducing digital barriers; specific strategies for facilitating the use of a digital application; specific strategies for facilitating the participation in virtual exercise groups; and strategies for facilitating external support, if needed. The priorities included improvement of digital literacy, assessment of training and technology support needs, technological support from family members, telephone feedback, personalized exercises, and exercise conducted in small groups.

Although mhealth interventions appear to be beneficial for increasing physical activity levels in OA (8, 30), there are still barriers to large-scale implementation, on personal, social, technological, and organizational levels (10). We present solutions for program adaptation that vary in complexity from single-component strategies to multifaceted and multilevel strategies (31). The variety of strategies proposed by our participants appears in line with the characteristics that m-health interventions for physical activity promotion should have (8, 30) and with the theoretical constructs for promoting and sustaining behavior change (BCT) (32). First, according to the existing literature, an essential strategy for increasing physical activity levels is to develop digital health-literacy training resources (8, 33). In our study, educational sessions, collaborative learning and paper or video guides represented priorities to improve self-efficacy and digital literacy at the individual, interpersonal and social/community levels. Second, for the participating health professionals, the assessment of access to digital infrastructure, social support and digital skills should be systematically and universally added to the comprehensive assessment of older people; this is a core element of AGILBcn (15) and is consistent with previous studies (34). Our findings are also consistent with the need for social and community support for the adoption and use of technology by OAs, for the resolution of technical problems, and for a decrease in the potential digital divide, as highlighted by other authors (8). This support might be provided by family members or by local networks (e.g., volunteers or peers).

Social interaction in face-to-face groups has been shown to benefit the adoption, increase and maintenance of physical activity (35, 36). In our study, older adults recognized virtual group delivery as an opportunity to remove some of the existing barriers to participating in face-to-face group programs and as a means to interact with peers, avoiding exposure to COVID-19. In contrast, CVS highlighted that the potential benefits of virtual delivery are undeniable, but that the pandemic has amplified the barriers to technology in OA, increasing their social isolation and loneliness (11). Controversy exists regarding the positive or negative impact of technology on loneliness, connectedness, and social support (33). Future interventions should seek to mitigate the social connectedness paradox of COVID-19 (37). Our groups emphasized the importance of combining non-digital alternatives to decrease the digital divide. Providing feedback is another important strategy to promote and maintain adherence to physical activity, and to trigger and sustain motivation for goal attainment (32). Among different options available [e.g., telephonic, *via* apps, wearable devices (38)], our participants still preferred to receive feedback by phone.

Attitudes of OA toward mhealth exercise vary (39). In our case, OA were willing to use technology-based exercise programs if they perceived them as useful or beneficial for achieving their goals. Interestingly, all participating groups paid little attention to safety and privacy in technology use, as compared to available evidence (10). OA focused on the adherence to and safety of home exercise performance, suggesting that technology is not an end in itself, but a mean.

While all the groups identified similar strategies, the results concerning priority and feasibility showed notable differences among groups. The OAs appreciated the limited size of participants in the virtual groups, the need for external support for participating in the intervention, personalization of the exercises, guarantee of access to technological support, incorporation of music in the virtual exercise sessions, and weekly telephone follow-up by HP. In contrast, significantly lower scores for these solutions were observed in CVS, and, in a smaller proportion, in HP.

CVSs scored lower on all items compared to the other two groups. In HPs, we observed a tendency to score higher for priority than for feasibility. This may be due to health professionals' experience regarding macro-, meso- and micro-level barriers to the implementation, scalability, integration and sustainability of mhealth interventions. Among the items for which HPs perceived feasibility to be higher than priority were: the use of messaging Apps (such as WhatsApp) to connect users, or as a vector for education; the creation of instruction booklets on the use of applications; and the incorporation of music in virtual exercise sessions. OAs perceived as a priority the design of an expert patient program and the sharing of information with family about the intervention, although ease of implementation was considered low, coinciding with HPs views. Conversely, the recruitment of local volunteers to provide support was deemed both a higher priority and a feasible step for all three groups.

We aimed to engage a wide range of stakeholders from an early stage to address the problem, identify strategies and prioritize them. This is in line with current policies, care practices and growing evidence on the importance of engagement and co-design for the development, implementation and adaptation of health promotion interventions and for the design of digital solutions (40, 41). However, there was some disparity in results regarding the potential benefits of this involvement concerning uptake and adoption (42). As in previous studies, the implementation of the co-design process was time- consuming, and it proved challenging to merge different stakeholder perspectives (43). In addition, involving OA in co-design was demanding, due to the extreme heterogeneity in physical and digital needs and capabilities (42).

As potential limitations of our study, the digital format of the AGIL Café sessions provided opportunities to participate in conversations during the COVID-19 pandemic, but was challenging due to technical limitations, such as signal loss, which resulted in certain segments in which the audio was missing (44). Although the platform allows the respondent to be seen, it is possible that we missed some non-verbal and body language cues, as participants often sit close to their cameras. Participants who were not technologically skilled required additional attention from the research team members, which led to a delay in the start of the sessions. The results should be interpreted with caution: the study was conducted in a particular area and with a particular group of participants, thus the results may not be completely generalizable; the integration of the different contributions made by the three different groups was limited to the final prioritization approach; and, finally, the phrasing of specific questions introduced the risk of being leading or suggestive (this, however, was necessary at the beginning of the sessions with OA, who had trouble understand more open questions).

As for strengths, the main advantage of the study was the combination of qualitative and quantitative research methods to provide complementary information. Another strength was the co-design approach involving all stakeholders, incorporating the diversity of perspectives of the AGILBcn program. Purposive sampling enabled us to recruit a wide range of participant types, although obviously selection bias cannot be completely excluded, as participation could have been skewed toward motivated individuals.

## 5. Conclusion

The present study provides practical solutions for implementing a technology-based, multicomponent program for older adults from a variety of perspectives, namely, those of older adults acting as end users, but also those of health professionals and professionals from the community. If confirmed by future studies in experimental and implementation research, these results might provide important considerations for policymakers, care providers, and practitioners, for designing, adapting, and implementing multicomponent, technology-based programs aimed to promoting physical activity in the older adult population. This can help to overcome barriers imposed by extreme conditions, such as the COVID-19 pandemic, and to improve adherence and enhance scalability to exercise programs.

## Data availability statement

The raw data supporting the conclusions of this article will be made available by the authors, without undue reservation.

## Ethics statement

The studies involving human participants were reviewed and approved by Clinical Research Ethics Committee (CREC) of the Foundation University Institute for Primary Health Care Research Jordi Gol i Gurina (IDIAPJGol) (20/048-P) and by the Ethical Commission of Animal and Human Experimentation (Authorization Number CEEAH 5066) of the Autonomous University of Barcelona. The patients/participants provided their written informed consent to participate in this study.

## Author contributions

LV-G, VD, LP, and MI contributed to the conceptualization of the study. LV-G, VD, LP, and LS-B performed the data collection. LV-G and VD were responsible for data curation and formal analysis. LV-G, VD, and MI were responsible for writing the initial draft. ER, PD, KK, MG-G, and CC-T reviewed and edited the draft. MI supervised the process of manuscript preparation. All authors agreed to be accountable for the content of the work. All authors contributed to the article and approved the submitted version.
