# Supplementary material for: Co-designing implementation strategies to promote remote physical activity programs in frail older community-dwellers
Source: Front Public Health. 2023 Mar 7;11:1062843. doi: 10.3389/fpubh.2023.1062843 (PMC10028273; doi:10.3389/fpubh.2023.1062843)
Supplement: Supplementary file 1 [file Data_Sheet_1.pdf]

Supplementary Material

Co-desing implementation strategies to promote remote physical activity programs in frail older community-dwellers

Lorena Villa-García*, Vanessa Davey, Laura Mónica Perez, Luis Soto-Bagaria, Ester Risco, Pako Diaz, Kerry Kuluski, Maria Giné-Garriga, Carmina Castellano-Tejedor, Marco Inzitari

***Correspondence:** Lorena.v22@gmail.com

Supplementary, Table 1. Initial script

Questions for older adults

- What would prevent you from exercising at home?
  - [Prompt] Is there anything that would concern you about doing the exercises at home?
  - If we provided you with instructions on exercises suitable for you, would it be helpful? How would you feel about doing these exercises?
  - Do you think you would be motivated or "find the time" to do the exercises every day?
  - Would it be helpful if a friend and/or family member were involved to support you?
  - What difference would it make if you knew you were going to receive follow-up calls from a physical therapist on a weekly basis, to see how you were doing with the exercise program?
  - Would you be interested in receiving calls from past +AGIL program participants and/or professionals involved in the program, in order to encourage and support you?
- Do you think using an app would help you do the exercises every day, or would it make it harder for you?
- Do you think you could do a series of exercises with a health professional via video call? (If not, why not)
  - [Prompt] What kind of support would enable you to feel more able to participate in physical exercise sessions via video call?
  - If you could participate once a week in a virtual group with a physical therapist (receiving the help you may in order to do so), do you think it would be more or less helpful to you than doing the exercises alone at home?

Questions for community stakeholders

- How can we reduce the barriers for older adults to participate in the virtual +AGIL intervention?
- What potential is there to support older people, through links with community initiatives or third sector agencies, to improve accessibility to the virtual +AGIL program?

Questions for health professionals

- What strategies could help reduce the barriers to older adults for participating in the virtual +AGIL intervention?
- Have you participated in any initiatives or strategies for solving some of these problems? (If so, what ideas were put forward? What was learned?)
- Do you have any recommendations for digital tools that would allow us to transfer the interventions proposed within the +AGIL Barcelona program, which include the promotion of physical activity and healthy diets, the prevention of loneliness and the monitorization of therapeutic complianc, to a digital format?

Supplementary, Table 2. Prioritization matrix template and scoring framework

**Prioritization category**	**Priority (P) and Feasibility (F) score**	**Interpretation**
Top priority, top feasibility	P= 75-100, F= 75-100	MUST DO/DO FIRST
Top priority, medium feasibility	P= 75-100, F= 50-74	MUST DO/DO FIRST
Medium priority, top feasibility	P= 50-74, F= 75-100	MUST DO/DO FIRST
Medium priority, medium feasibility	P= 50-74, F= 50-74	IMPORTANT/ DO SECOND
Top priority, low feasibility	P= 75-100, F= 25-49	IMPLEMENT IF POSSIBLE
Low priority, top feasibility	P= 25-49, F= 75-100	IMPLEMENT IF POSSIBLE
Medium priority, low feasibility	P= 50-74, F= 25-49	BEAR IN MIND/ CONSIDER
Low priority, medium feasibility	P= 25-49, F= 50-74	BEAR IN MIND/ CONSIDER
Top priority, no feasibility	P= 75-100, F= 0-24	DO NOT CONSIDER
Medium priority, no feasibility	P= 50-74, F= 0-24	DO NOT CONSIDER
Low priority, low feasibility	P= 25-49, F= 25-49	DO NOT CONSIDER
Low priority, no feasibility	P= 25-49, F=0-24	DO NOT CONSIDER
No priority, top feasibility	P= 0-24, F=75-100	DO NOT CONSIDER
No priority, medium feasibility	P= 0-24, F=50-74	DO NOT CONSIDER
No priority, low feasibility	P= 0-24, F=25-49	DO NOT CONSIDER
No priority, no feasibility	P= 0-24, F=0-24	DO NOT CONSIDER
